# Emotional Regulation, Sensory Over-Responsiveness and Developmental Coordination Disorder in Midlife

**DOI:** 10.1093/geroni/igaf122.961

**Published:** 2025-12-31

**Authors:** Merav Asher, Maayan Agmon

**Affiliations:** University of Haifa, Haifa, HaZafon, Israel; University of Haifa, Haifa, HaZafon, Israel

## Abstract

Developmental Coordination Disorder (DCD) is a prevalent neurodevelopmental condition that causes lifelong challenges across all aspects of life. Research has predominantly examined DCD in children, establishing connections with sensory over-responsiveness (SOR) and emotional dysregulation. However, evidence regarding these relationships in midlife adults remains unexplored. This study investigated the relationships between SOR and DCD in midlife, considering the role of emotion regulation, highly relevant for healthy aging, as a mediator. Data were collected from 89 middle-aged adults (mean age 45.59±0.68; 47% women) using the Adult Developmental Coordination Disorders/Dyspraxia Checklist (ADC), Sensory Responsiveness Questionnaire Intensity Scale (SRQ-IS), and Difficulties in Emotion Regulation Scale (DERS). A mediation analysis examined the role of emotional dysregulation in the relationship between SOR and DCD symptoms. Results showed that SOR significantly predicted DCD symptoms (β = 0.303, p = 0.006). However, when emotional dysregulation was included, the direct effect of SOR on DCD symptoms became non-significant (β = 0.106, p = 0.278), while the indirect effect via emotional dysregulation was significant (β = 0.197, p < 0.05, 95% CI [0.082, 0.343]). This finding suggests complete mediation, indicating that SOR increases emotional dysregulation (β = 17.971, p = 0.001), which in turn exacerbates DCD symptoms (β = 0.011, p < 0.001). Our findings demonstrate a developmental shift wherein emotional regulation becomes the primary pathway linking sensory processing to motor coordination in midlife, rather than the direct sensory-motor connections observed in childhood. This suggests that interventions targeting emotional regulation may be more effective than those focusing solely on sensory processing to address persistent coordination difficulties in midlife adults.

